# Notes and Notices

**Published:** 1889-09

**Authors:** 


### NOTES AND NOTICES.

Removals.—Dr. J. J. Sturgus, from Olathe, Kan., to Seattle, Washington Territory; Dr. C. E. Dennis, from Thurlow, Penna., to Bayonne, New Jersey; Dr. L. H. Lemke, from St. Louis, Mo., to De Soto, Jefferson County, Mo ; Dr. B. Bryant, from Gilroy, Cal., to San José, Cal.; Dr. W. H. Ross, from St. Louis to 727 Lexington Street, Louisville, Ky.; Dr. William S. Gee, from Hyde Park, Ill., to 5401 Jefferson Avenue, Chicago; Dr. S. Mills Fowler, from St. Augustine, Fla., to Dallas, Texas; Dr. C. S. Durand, from New York City to Mungeli, Central Provinces, India; Dr. Edgar R. Bryant, from Philadelphia to Hahnemann Hospital, 4th Avenue, New York City, where he takes the position of resident physician. Dr. John Dike has succeeded to the practice of Dr. McIntosh at Melrose, Mass., whilst Dr. McIntosh has succeeded the late Dr. Keith in the latter's practice at Newton, Mass. Dr. C. O. Boyce has settled at Ishpeming, Michigan.

The New Board of Censors of the I. H. A., Dr. S. A. Kimball informs us, has been incorrectly reported in our pages. It should read: Drs. Schmitt, Bell, Wesselhœft, Rushmore, and Dillingham.

The Homœopathic Medical Society of the State of Pennsylvania will hold its annual meeting at Pittsburg on the 17th, 18th, and 19th of September. The Allegheny County Society will be glad to welcome all physicians, and will entertain them royally. A large number of good papers are already announced. It is desired to make this meeting a specially brilliant and memorable one. The Old School hold their annual meeting in Pittsburg about the same time our gathering will be held; comparisons will be made.

Arrangements have been made with the Pennsylvania Railroad Company by which reduced rates to Pittsburg and return can be obtained by delegates to and members of the State Society. Excursion tickets will be good from the 14th to the 21st of September. The reduced rates (two cents per mile) can only be secured by procuring a card order from the Corresponding Secretary, Edward R. Snader, M. D.

The Indiana Institute of Homœopathy will hold its twenty-fourth annual session at Indianapolis, in May, 1890. The President is Dr. J. F. Thompson, of New Castle; Vice-President, Dr. E. W. Sawyer, of Kokomo; Treasurer, Dr. J. S. Martin, of Muncie; Secretary, Dr. Wm. B. Clarke, of Indianapolis. In the circular of announcement, the Secretary says:

"At the 1889 meeting it was decided that special efforts should be put forth to make the 1890 session far surpass that of any previously held, in point of interest and attendance, and to this end a special Booming Committee was appointed to consider how best to achieve this result, with the special recommendation that it might be advisable to make the affair attractive and interesting to the whole family by extending special entertainment to members, their friends and patrons, with their wives or female accompaniments, and also that it will be necessary for each member to do his direct, individual duty, and, in addition, work among his or her friends to increase the membership in the Society and the interest in the work being done by it. The Booming Committee is composed of Doctors Taylor, of Crawfordsville; Runnels, of Indianapolis; Bowen, of Fort Wayne; Sawyer, of Kokomo, and Thompson, of New Castle, and the regular Committee of Arrangements for next year is made up of Doctors Compton, Clemmer, and Runnels, all of Indianapolis.

To Builders and those who Contemplate Building.—One of the most useful publications for builders and persons contemplating building is the beautifully illustrated *Architect and Builder* edition of the *Scientific American*, published monthly by Munn & Co., the celebrated Patent Solicitors, at 361 Broadway, New York.

It has become the custom of most of the builders of the United States and Canada to keep on file this publication, not only for their own benefit, but for the use of their customers, and they find their business promoted by so doing. A great variety of dwelling-houses, costing from a few hundred to several thousand dollars, are illustrated in each monthly number, besides a double page printed in colors, representing one or more handsome residences already built. After the design for the elevation or style of the house has been selected, builders are enabled to give a close estimate of the cost of construction, as the working plans accompany the elevation. Most persons contemplating the building of a house or stable for their own use derive both pleasure and considerable saving, sometimes, by carefully considering at their leisure, and by their fireside, various designs and plans which may come before them. To enable a person to come to a wise conclusion in such an important matter as building a home for his family, he will be wise if he brings the subject before his entire household, and studies carefully over in the domestic circle the style of house and the interior arrangements. It not only affords great pleasure to the entire family to be considered in the matter, but good suggestions will come from it, and mistakes will be less likely to occur in the selection. By all means consult the wife and grown-up daughters, if so fortunate as to have them, and to this end everybody who contemplates building should provide himself with a complete file of the *Architect and Builder* edition of the *Scientific American*, some forty numbers, and then he will have at hand not only the best material to select his design from, but he will also find the publication useful and profitable to refer to while the building is being constructed.

If a person does not find the design for a house, or other structure he contemplates building, that suits his fancy, or the estimate of the cost is too great, in a single number of the publication, he will be very sure to find in some one of the other numbers something that will suit both his fancy and purse. Hundreds of dwellings have been erected on the plans that have appeared in this publication, and any person who contemplates building, or who wishes to alter, improve, extend, or add to existing buildings, whether wings, porches, bay windows, or attic rooms, will be pretty sure to find what he wants in the *Scientific American Architect and Builder*, which is published on the first of each month, at the office of the *Scientific American*, 361 Broadway. Subscription price, $2.50 a year, twelve numbers. Single copies, 25 cents. Back volumes of six numbers, in flexible covers, in imitation of Turkey Morocco, $2.50. Subscriptions received and volumes sold by all newsdealers.

I. H. A. Notice.—Attention is called to the fact that no application for membership will be accepted, unless an original thesis, consisting either of an original proving or a clinical report of three cases, is handed in before March 1st, 1890. At the last meeting of the I. H. A., six applications were rejected, because no theses were presented with them, and this rule will be strictly adhered to.

Applications for membership should be sent to the Chairman of the Board of Censors, Dr. Julius G. Schmitt, 113 North Ave., Rochester, N. Y., on or before December 1st, 1889, and theses must also be sent to him, not later than March 1st, 1890. The next meeting of the I. H. A. will be held at Newport, R. I.; the date and other particulars will be announced later.

S. A. Kimball, *Secretary.*

What are the Remedies?—In our June No., page 258, we gave a list of symptoms, the answers to be guessed by our readers.

We now announce the answers: No. 1, Cimex; No. 2, Cimex; No. 3, Hypericum; No. 4, Ipecac.; No. 5, Ipecac.; No. 6, Iris; No. 7, Iris; No. 8, Kreosote; No 9, Ledum; No. 10, Oxalic acid. Four of our subscribers have answered. They are as follows: Dr. John V. Allen, Frankford, Philadelphia; Dr. H. P. Holmes, Sycamore, Illinois; Dr. A. Kilmer, Gibson, Tennessee; Dr. George W. Dunn, Atlanta, Illinois. All have answered correctly, except that Dr. Dunn gives for No. 4 Ipecac. and Lycopodium.

Dr. Holmes remarks: "The most of these are found in Hering's *Condensed;* but a few of them are found only in *The Guiding Symptoms.* Only one was found by using a repertory."

The October and November Numbers.—We call special attention to the notice on second page of cover for this month. It will be seen that we intend to issue another chapter of the repertory. As it is so extensive that it will include two numbers of the Journal, we have, therefore, decided to include the October and November numbers in one issue. There will, therefore, be no October number, separately published. The consolidated number will be issued as soon in November as possible. We repeat this statement here, lest the other notice on the cover escape the attention of our readers.

